# Core, Coating, or Corona? The Importance of Considering Protein Coronas in nano-QSPR Modeling of Zeta Potential

**DOI:** 10.1021/acsnano.2c06977

**Published:** 2023-01-18

**Authors:** Selvaraj Sengottiyan, Alicja Mikolajczyk, Karolina Jagiełło, Marta Swirog, Tomasz Puzyn

**Affiliations:** † Laboratory of Environmental Chemoinformatics, Faculty of Chemistry, University of Gdansk, Wita Stwosza 63, Gdansk80-308, Poland; ‡ QSARLab, Trzy Lipy 3, 80-172Gdansk, Poland

**Keywords:** polymeric nanoparticle, coating, protein-corona formation, zeta potential, nano-QSPR, machine learning, GA-PLS, advanced nanomaterials design, SSbD

## Abstract

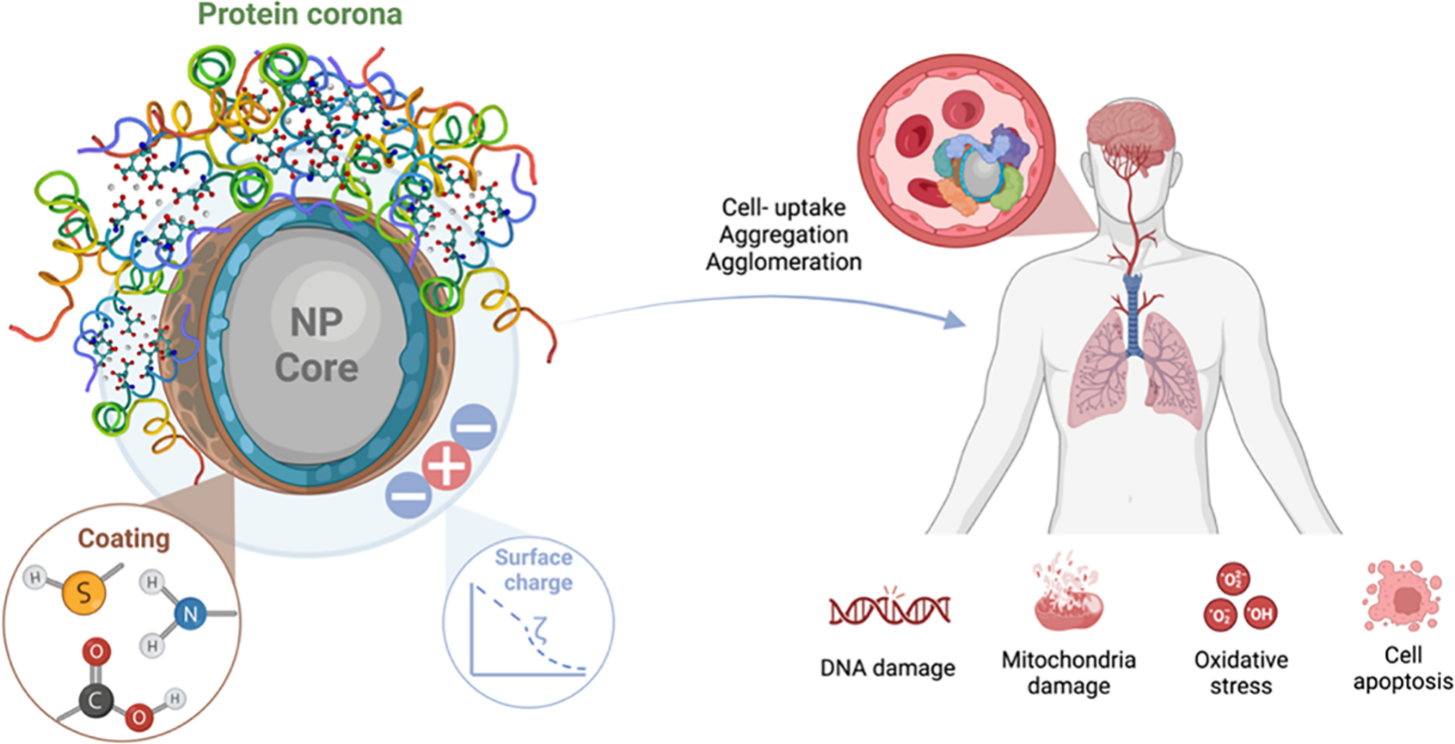

To control stability in a biological medium, several factors affecting the zeta potential (ζ) of nanoparticles (NPs) must be considered, including complex interactions between the nanostructure and the composition of the protein corona (PC). Effective *in silico* methods (based on machine learning and quantitative structure–property relationship (QSPR) models) could help predict and characterize the relationship between the physicochemical properties of NP and the formation of PC and biological outcomes in the medium at an early stage of the experiment. However, the models currently developed are limited to simple descriptors that do not represent the complex interactions between the core, the coating, and their PC fingerprints. To be useful, the models developed should be described as a function of both the structural properties determined by the core and coating of the NPs and the biological medium determined by the formation of the protein corona. We have developed a set of complex descriptors that describe the quantitative relationship between the value of the zeta potential (ζ), core, the coating of NPs, and their PC fingerprints (the so-called nano-QSPR model). The nano-QSPR model was developed based on a genetic algorithm using a partial least-squares regression method (GA-PLS), which is characterized by high external predictive power (*Q*^2^_EXT_ = 0.89). The GA-PLS model was developed using descriptors that describe (i) the core structure (determined by 7 different types of polymer-based NMs in the range of 20 different sizes), (ii) the coating structure with 7 different functional groups, and (iii) 80 different types of protein compositions adsorbed on the surface of the NPs. The presented study answers the question of how complex interactions between the corona and NP determine the zeta potential (ζ) of NP in a given medium. Moreover, our current study is a proof-of-concept that the zeta potential of NPs modeled on the original structure depends not only on the NPs themselves but also on the structure and properties determined by the NP core and coating, as well as the biological medium determined by the formation of the protein corona. On the basis of these results, our studies will be useful in determining the stability and mechanism of cell uptake, toxicity, and ability to predict the zeta potential of compounds not yet tested.

## 1 Introduction

**Figure 1:**
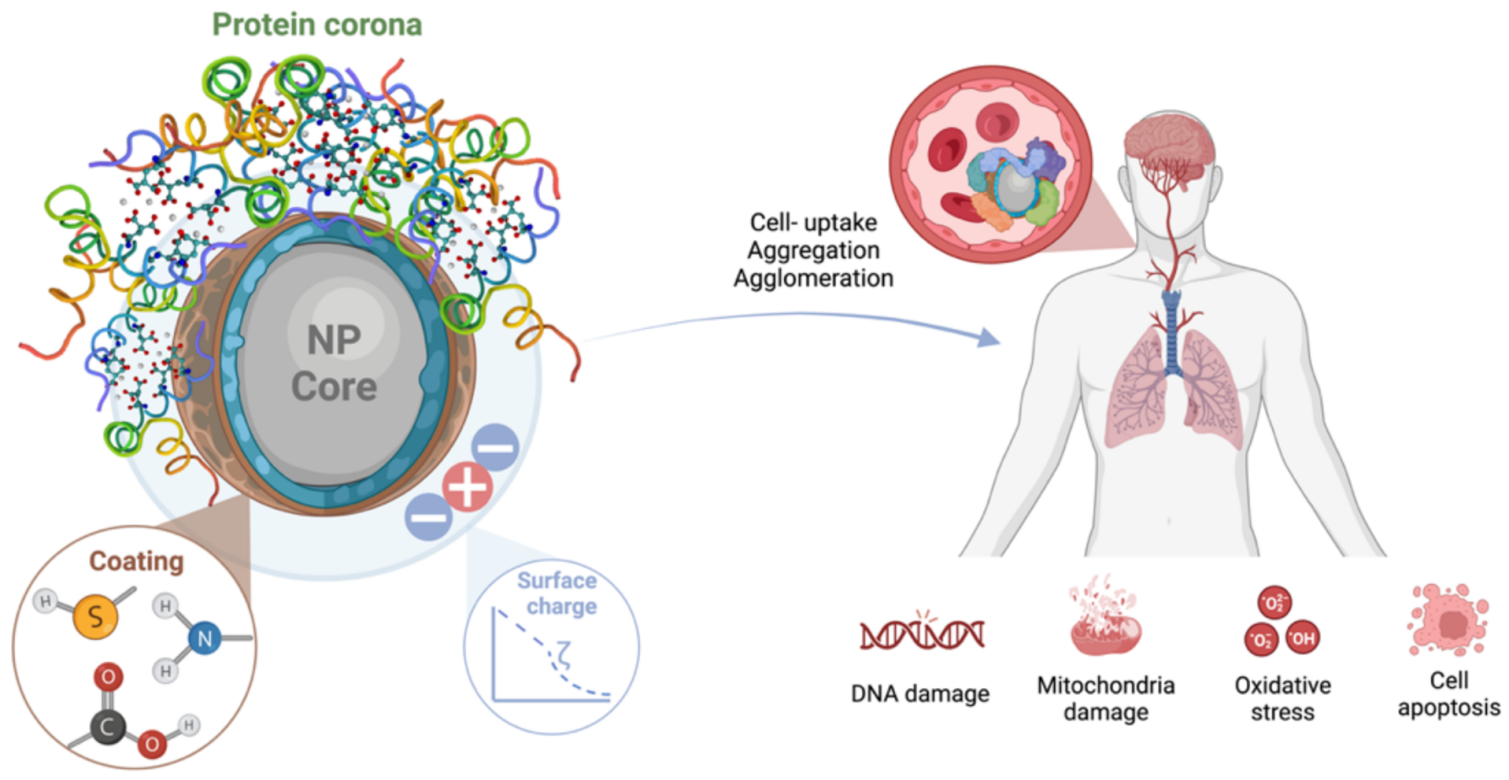
Formation of protein corona is caused by changes in NP structure and zeta potential (ζ) that lead to changes in cellular uptake (aggregation or agglomeration).

The zeta potential (ζ) is a parameter commonly used to determine the stability of the suspension (a tendency to determine the aggregation or agglomeration process of nanoparticles) and the surface morphology of nanoparticles (charge in the diffuse layer).^1a−1e^ Knowledge of the zeta potential (ζ) is crucial for studying the cellular uptake of NPs. Therefore, this parameter is widely used in nanotoxicology for NP characterization, NP surface bioactivity, and safety assessment. However, the structure of NPs can change during their lifetime and is highly dependent on the suspension (e.g., biological medium). Therefore, the zeta potential (ζ) is a property that includes the particle itself and its environment-dependent interaction (NPs with the protein environment). For example, depending on the dispersion system (biological medium), concentration, pH, and NPs, proteins can be adsorbed on the surface.^2^ The “protein coronae” formed from the adsorbed NPs can significantly alter the surface properties (see Figure 1) that affect the biological behavior of the NPs. This can lead to changes in the stability of the dispersion and in its functionality, which sometimes decreases or improves the functionality of NPs, resulting in loss or gain of function, which subsequently can also determine changes in the toxicity of NPs (e.g., biodistribution, uptake, opsonization, kinetics).^3a,3b^ For example, NPs can agglomerate or aggregate. When the organism/cell ingests the aggregated/agglomerated NPs, depending on the environment, such as pH, concentrations, and protein corona, the NPs may dissociate into smaller particles. Decreasing the size of NPs can lead to an increase in surface area. Thus, NPs in the dispersion can behave like a “Trojan horse” that becomes more toxic to the human body and the environment.^2^ The behavior of NPs can be controlled by the surface charge, which stabilizes the dispersed NPs in suspension and eventually prevents them from aggregation or agglomeration. With the available experimental techniques, the surface charge can be measured directly. The surface charge can be estimated based on a measured zeta potential value (ζ) in a given biological medium.^4^ In the literature, numerous studies have attempted to experimentally characterize the zeta potential (ζ) of NPs.^5^ However, experimental evaluation of NP interactions with biological systems and measurement of their zeta potential (ζ) is expensive and time-consuming. Therefore, the application of faster and less expensive chemoinformatic methods that can support the characterization of the zeta potential (ζ) value for different NPs is of high interest.^3−6^ For example, Lobaskin et al.^7^ applied a hybrid MD/LB simulation method to study colloidal electrophoresis and an effective dynamic charge of colloid^8^ (i.e., the value of the zeta potential (ζ)). The study by Varsou et al.^9^ demonstrates the application of a read-across method for predicting the NM zeta potential using a set of image descriptors derived from transmission electron microscopy images (TEM) of the NM as input data. A similar study was provided by Papadiamantis et al.^10^ In the study,^10^ authors used a set of molecular descriptors that can be easily acquired or calculated using atomic periodicity and other fundamental atomic parameters to develop a predictive in silico model for the ENM zeta potential of 68 ENMs. Mikolajczyk et al.^3a^ applied the quantitative nanostructure–property relationship approach (nano-QSPR) together with nanostructure descriptors to predict the zeta potential of various metal oxide nanoparticles.^3a^ The results presented by Mikolajczyk et al.^3a^ indicate that the experimentalists may successfully apply computational methods to predict the relationships between the zeta potential (ζ) and the structural characteristics of different types of nanoparticles. In 2016, Wyrzykowska et al.^3b^ applied a developed nano-QSPR methodology to predict the zeta potential of different metal oxide nanoparticles determined by the action of an ionic solution (KCl).^3b^ However, the zeta potential data derived from the experimental measurement are inconsistent. Thus, developing a predictive computational model that may characterize the impact of zeta potential is an important challenge. Recently, Siozochenko et al.^6^ proposed a data curation framework to curate the quality of zeta potential data sets to make them useful for computational modeling. In article,^6^ the authors provided a structure–property relationship (nano-SAR) model to predict the values of zeta potentials for nanoparticles measured in different media (e.g., pH, presence of ions (Na^+^, K^+^), culture medium (RPMI-1640, DMEM, Holtfreter medium, etc.). However, there is still a lack of rapid methods to predict the relationship between zeta potential (ζ), nanostructure properties, and the toxicity of untested NPs. Therefore, computational methods have been introduced to predict the quantitative relationship between the value of the zeta potential (ζ) and the structure of NP.^11^ Theoretical models have confirmed that the zeta potential (ζ) is affected by the structure of NP (intrinsic particle properties, such as size or concentration).^2,12^ Unfortunately, these models do not take into account additional factors such as suspension conditions, pH, temperature, and ionic strength, which may affect the value of zeta potential, so the stability of NPs differs when serum is introduced into the cell culture medium (the tendency of NPs to aggregate or agglomerate). Serum proteins may prevent NP agglomeration after dispersion or cause agglomeration by adsorption of serum proteins on the surface.^13^ However, there is a lack of knowledge about the effects of different surface modifications on the quantitative/qualitative composition of the corona and their influence on the zeta potential, which determines the stability and agglomeration/aggregation of functionalized NPs in a given medium. In addition, little is known about the effects of a specific corona composition on the uptake and associated toxicological profile of NPs. Therefore, the development of strategies for classical computational models that can determine the relationship between zeta potential (ζ) and NP core, coating, and corona is among the most challenging and important tasks for computational nanotoxicologists.

To address this challenge, we applied the integration of machine learning methods based on the Genetic Algorithm Partial Least Square (GA-PLS) and nanodescriptors that determine both the intrinsic and extrinsic properties of NPs would greatly enhance the potential of *in silico* methods to predict the zeta potential of NPs in a given biological medium and correlate well with experimental predictions.^14^ However, GA-PLS and GARGS work similarly; only chromosome optimization is different. In GARGS,^15^ using comparative molecular field analysis (CoMFA) expressed in terms of computer graphics of digital differential analysis (DDA) of spatial arrangement, but in the case of GA, the type of encoding of the chromosome is the optimization of descriptors based on the *R*^2^ value of selecting the best score for selecting descriptors. Based on this application, our problem of interest (GA-PLS) method would be sufficient. In addition, we developed a predictive nano-QSPR model that describes the relationship between the structure of polymeric nanomaterials represented by the core, coating, corona properties, and zeta potential in biological media.

## 2 Results and Discussion

By combining the genetic algorithm with the PLS method (GA-PLS), the nano-QSPR model was developed to quantitatively describe the value of the relationship between the zeta potential (ζ) and the core, coating, and protein corona of 20 PNP (eq 1, Table 1, Supporting Information, Table S3).
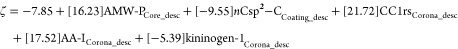
1

**Table 1:** Data Set of 20 PNPs Used for the Development of the nano-QSPR Model.

	nanoparticle structure		core descriptor	coating descriptor	corona			zeta potential (end point)		
structure ID	core	coating	AMW-P	*n*Csp2–C	complement C1r subcomponent	Apo A-I	kininogen-1	observed	predicted	data split
**5**	polystyrene	carboxy functional	6.06	1	0	0	1	–70	–59	T
**4**	polystyrene	carboxy functional	6.06	1	0	0	0	–60	–59	T
**18**	poly(isobutylcyanoacrylate)	heparin coated	6.35	3	0	1	0	–50	–35	V
**2**	polystyrene	carboxy functional	6.06	1	0	1	0	–30	–34	T
**11**	polystyrene	polyglycerol	6.06	0	0	0	0	–30	–26	T
**15**	poly-ε-caprolactone	dextran-coated (D/DC)	5.96	1	0	1	0	–20	–16	V
**19**	poly(hexadecyl cyanoacrylate)	polyethylene glycol	5.34	0	0	1	0	–20	–10	T
**17**	poly(isobutylcyanoacrylate)	dextran-coated (D/DC)	6.35	1	0	1	0	–15	–19	T
**3**	polystyrene	carboxy functional	6.06	1	0	1	0	–10	–22	T
**14**	poly-ε-caprolactone	polyethylene glycol	5.96	0	0	1	0	–10	2	V
**16**	poly-ε-caprolactone	dextran-coated (D/DC)	5.96	1	0	1	0	–5	–16	T
**1**	polystyrene	carboxy functional	6.06	1	0	1	0	–4	–22	V
**6**	polystyrene	amino functional	6.06	0	0	1	0	5	20	T
**9**	polystyrene	amino functional	6.06	0	0	1	0	5	8	T
**8**	polystyrene	amino functional	6.06	0	0	1	0	10	8	V
**10**	polystyrene	amino functional	6.06	0	0	1	0	10	8	T
**13**	poly(lactic-*co*-glycolic acid)	didodecyldimethyl-ammonium	8.23	0	0	0	0	15	7	T
**20**	poly(glycidyl methacrylate)	polyethylene glycol	6.98	0	0	1	0	20	15	V
**12**	poly(lactic-*co*-glycolic acid)	didodecyldimethyl-ammonium	8.23	0	0	1	0	45	45	T
**7**	polystyrene	amino functional	6.06	0	1	0	0	50	49	T

The variance in molecular structure is expressed in the model by two latent vectors (LV1 = 53.1%, LV2 = 36.1%), which are linear combinations of five descriptors corresponding to the core structure (i.e., the atomic molecular weight, *AMW-P*), the structure of the coatings (i.e., the number of hybridized carbon atoms, *n*Csp^2^–C), and the structure of the protein corona (expressed by (i) a serine protease) joining with C1q and C1s to form C1, the first component of the classical pathway of the complement system - complement C1r subcomponent, CC1rs; (ii) alpha-amylase inhibitor, AA-I; (iii) alpha-2-thiol proteinase inhibitor, kininogen-1), as shown in Table 1. The GA-PLS model (eq 1) was developed according to the guidelines of the recommendations of OECD-QSAR.^16^

**Figure 2:**
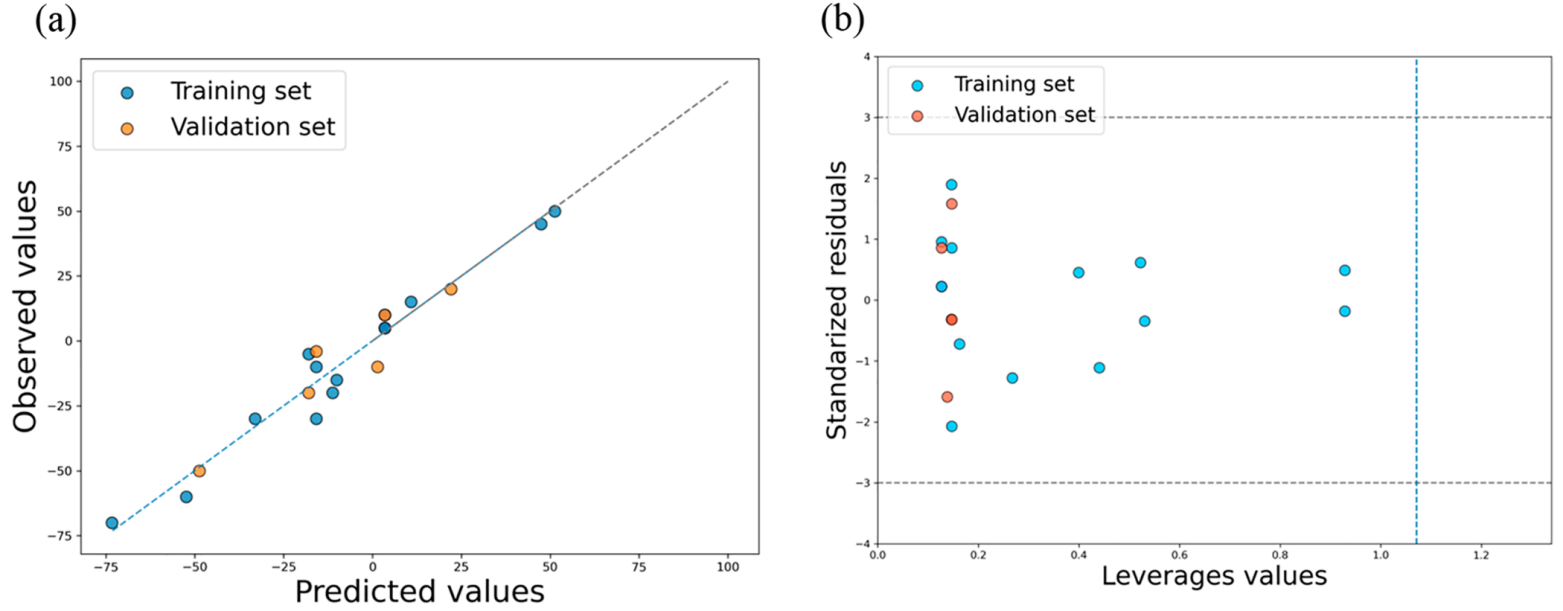
(a) Plot of experimentally observed and predicted zeta potential values for training and validation compounds for nano-QSPR models; (b) Williams diagrams for the nano-QSPR model.

The optimal combination of descriptors and the number of LVs were selected based on the results (i.e., the lowest value of RMSE_CV_ = 24.509). The two LVs together explained 89.2% (LV1 = 53.1%, LV2 = 36.1%) of the structural variance (in the descriptors) and 95.7% (LV1 = 83%, LV2 = 12.5%) of the variance in the zeta potential (ζ). The predictive power of the model (*Q*^2^) corresponds to 89.4% of the covariance, with RMSE_P_ = 7.331. The statistical measure of the coefficient of determination of covariance *R*^2^ = 95.7% indicates a better fit in the regression analysis. The plot of experimental values versus predicted values (Figure 2a) showed very good agreement between the observed and predicted zeta potential values for the 20 PNPs in both the training and validation sets, confirming the predictive capacity of the developed model.

The applicability range of the best model was evaluated using the Williams diagram (Figure 2b). In the analysis of the residual values (*y*_obs_–*y*_pred_) for the training and validation rates of the compounds, no value is above the residual value of 3 standard deviations from the average residual. Furthermore, none of the structures of the studied PNPs differed significantly from those of the nanoparticles of the training set; all were characterized by the leverage values *h* < h* = 1.07. From the above observation, this model can be successfully applied to predict the zeta potential of all tested PNPs and to predict untested PNPs when the calculated value is below the critical value (h* = 1.07).

**Figure 3:**
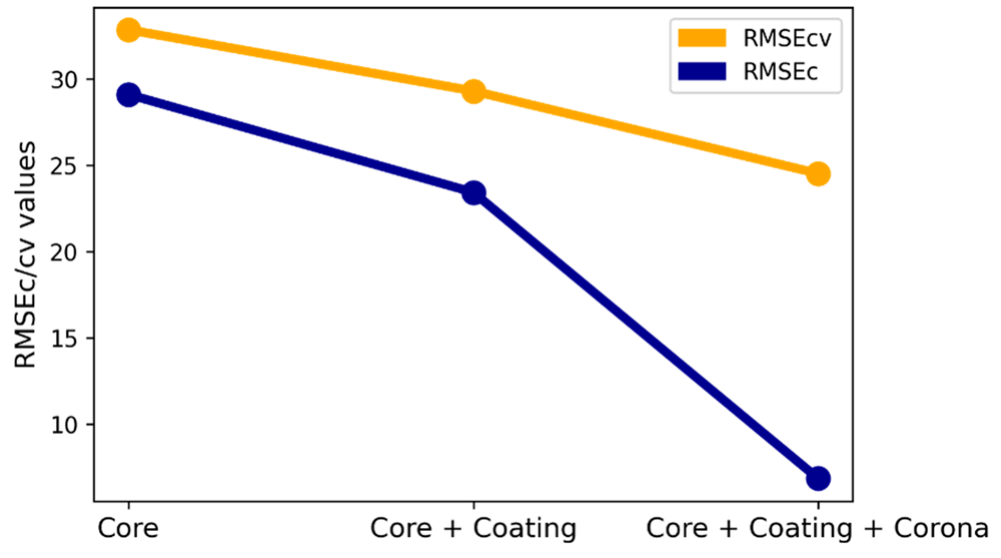
REMSEc/cv values for three different domains.

Depending on the problem, we have modeled three different domains, namely, the core, the core + coating, and the core + coating + corona, respectively. Based on our problem, we will determine which part of the domains plays a crucial role in influencing the zeta potential to a large extent, and for that, we will define this concept. We applied the root-mean-square error calibration/cross-validation methods (see Figure 3). It shows that there is less error in the values of the domains, which gives a more fit prediction based on this analogy, and we could strongly suggest that the zeta potential is mainly influenced first by the core + coating + corona domains and then second by the core + coating domain and the last of the core domains.

**Figure 4:**
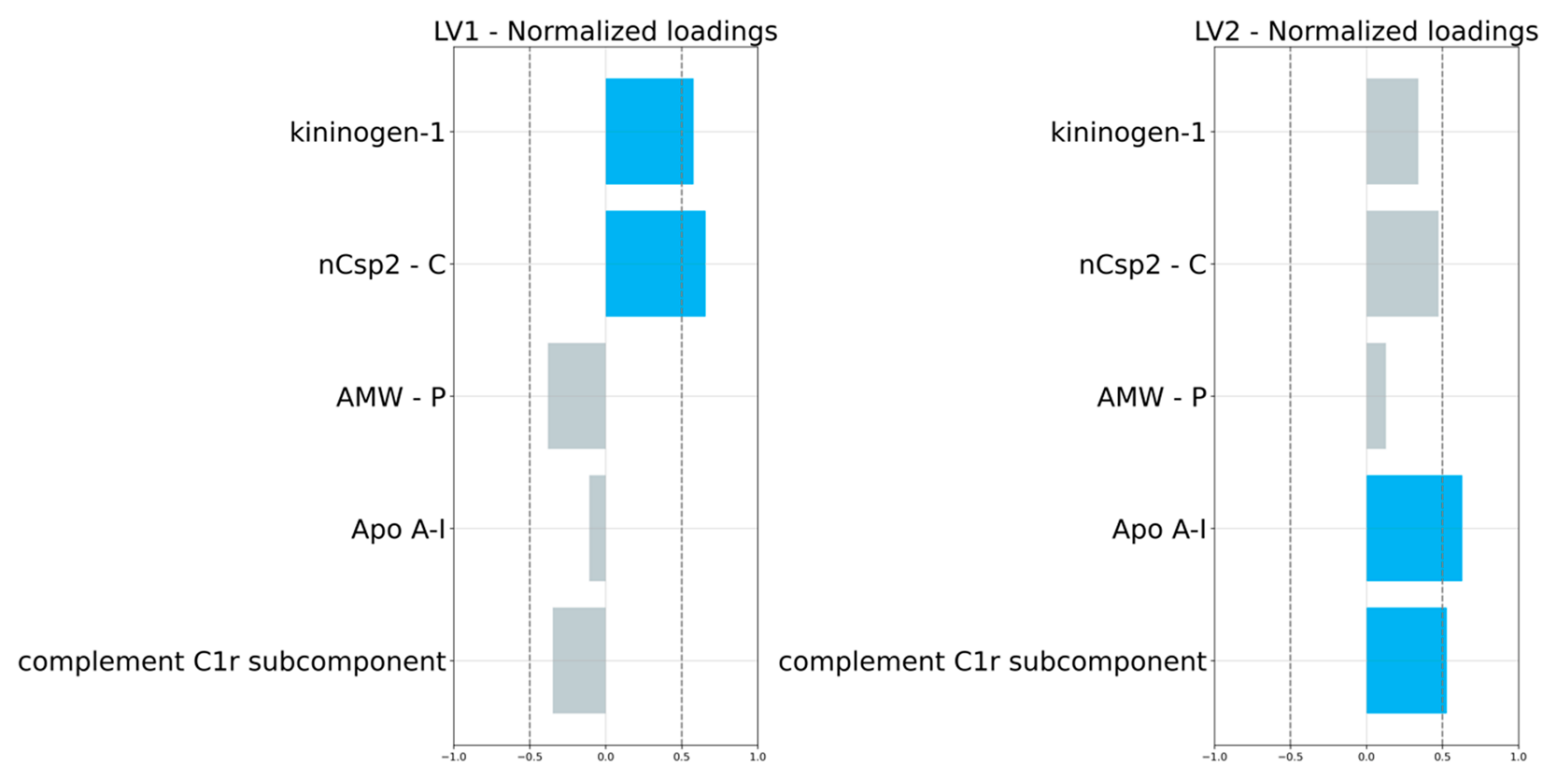
Loading values of individual latent vectors (LVs).

Considering the loading values, the first and second latent vectors (LV1, LV2) are mainly associated with kininogen-1, *n*Csp2–C, AMW-P, Apo A-I (AA-I), and complement C1r subcomponent (CC1rs), as shown in Figure 4.

### 2.1 Mechanistic Interpretation of the Developed nano-QSPR Model

The nano-QSPR model (eq 1, Table 1, Supporting Information, Table S3) is based on the combination of several important descriptors: one descriptor that describes the core structure (AMW-P), one characterizing the coating (*n*Csp^2^–C), and three descriptors that correspond to the formation of the protein corona (i.e., CC1rs, AA-I, and kininogen-1, respectively). The importance of the core and corona descriptors represented by AMW-P (standardized coefficient: 16.228), CC1rs (coefficient: 21.720), and AA-I (protein descriptors, standardized coefficient: 17.520) is about two times higher than the coating descriptor (*n*Csp^2^–C, standardized coefficient: −9.559, eq 1, Table 1). Thus, the presented results clearly show that the zeta potential (ζ) is determined by the structure defined as core and coating (AMW-P, *n*Csp^2^–C, eq 1, Table 1) and by the protein corona (described by the descriptors denoted by CC1rs, AA-I, kininogen-1, Table 1). However, there is very limited information in the literature and a lack of computational models depicting the correlation between the core structure, the coating, the corona, and the zeta potential value of NPs.^10^

### 2.2 Characterization of Nanoparticles Based on Physicochemical Properties and PC Adsorption

According to the usual criteria, the particles are very stable in solutions with a zeta potential in the range ζ < −30 mV or > + 30 mV.^17^ When the values of the zeta potential (ζ) tend to be 0, the possibility of dispersion is very limited, which leads to the fact that the phenomenon of agglomeration/aggregation occurs easily. Of the 20 samples, 15 samples (PS_CF_2, PS_Pol, PCL_D_5, PHDCA_PEG, PIBCA_DC, PS_CF_3, PCL_PEG, PCL_D_40, PS_CF_1, PS_AF_1, PS_AF_4, PS_AF_3, PS_AF_5, PLGA_DA_2, and PGMA_PEG) were reported to have values close to ζ > ± 30 mV (Table 1). The PS-NH_2_ sample had the most positive zeta potential (ζ) equal to +50 mV. Similarly, PLGA-dido-decyl-dimethylammonium has a zeta potential of (ζ) equal to +45 mV. We hypothesize that these two compounds are more likely to absorb cells due to the stronger cationic surface charge of NP than the anionic surface charge of NP, which has the electron-donating property of the amino group (NH_2_) due to the combination of PS-NH_2_, a positively (+) charged NP. Similarly, this combination of PLGA-dido-decyl-dimethylammonium; this phenomenon strongly correlates with the experimental data.^18^ Based on the value of ζ, we can speculate that functional groups (coatings) play an important role in influencing the structure and properties of NP. This influences the ζ value.^1e^ On the basis of these values, the nanoparticles coated with acidic functional groups are in the range of negative zeta potential and vice versa, and those with basic functional groups are in the range of positive zeta potential. In our case, regarding protein adsorption, the combination of PS_CF1 (150 nm) nanoparticles has a higher mode than others, and a similar combination of PS_CF2 (130 nm) and PS_CF2 (140 nm) compounds has lower adsorption on the surface. This is because, based on a recent report,^20^ this combination (PS_CF1) of nanoparticles has a large surface area relative to volume with a higher order of magnitude diameter and that these functionalized nanoparticles remain in the suspended solution even at higher concentration. However, this is still controversial with the concept^21^ of negative zeta potential (−4 for PS_CF1), which can adsorb protein that is sometimes absorbed very little or not at all, which is still an unclear phenomenon in our case. The combination of PS_AF-functionalized nanoparticles contributes to the second- and third-largest protein adsorption due to the charge^22^ and many interactions of these nanoparticles. The combination of PCL_D_40 and PGMA_PEG nanoparticles is the last in adsorption with proteins, perhaps because of the more complex structure.

### 2.3 Influencing the Zeta Potential by Polymer-Coated NP with Protein Corona

Knowledge of the protein corona formed after contact with NPs plays a critical role in determining the zeta potential and, consequently, in characterizing the nature of NPs, including clearance of the mesosystem, biological fate, opsonization, or uptake of NPs^23^ (Figure 1). Experimental studies confirm^1b,1c,12,24^ that these dynamic processes associated with corona formation can influence zeta potential (ζ), cellular uptake, and NP fate of NPs in the body, determining cellular responses, such as cytotoxicity.^24^ For example, Vroman and Lukosevicius^1d^ demonstrated in the 1960s that the interaction of NPs with any biological medium leads to the formation of a protein corona. The arrangement of proteins that attach to the nanovectors depends on many factors, including (i) the properties of the NPs (including material type, size, and surface charge) and (ii) the composition of the biological substrate. Consequently, the protein coating imparts a biological identity to the NPs, determining their stability, biological distribution, interactions, and toxicity. This identity can be determined by the zeta potential (ζ) and the pH of the biological medium.^25^ On the basis of the PNP characteristics, the composition of the biological medium in the developed nano-QSPR model (eq 1) is expressed by the core descriptors (AMW-P), the coating (*n*Csp2–C), and corona (CC1rs, AA-I, kininogen-1) (Table 1). The results of the developed model (eq 1) clearly show that modelers should go beyond “average” and “standard” nanostructure descriptors when developing true models to provide key properties of true NPs (i.e., descriptors describing their temporal heterogeneity in the environment). One of the most common factors that determine NP heterogeneity over time (e.g., NP uptake into the cell and between cellular compartments in the biological system) is the formation of a protein corona. The time-resolved protein corona could be treated as NP fingerprints (system-dependent descriptors) that capture its evolution based on binding affinities and local abundances in response to physiological signals.

### 2.4 Selective Roles of NP with Protein Descriptors

The FPs used in the study encode the structural information within a molecule as a bit vector. They are a sequence of bits, where a bit equal to 0 indicates the absence of a structural protein feature corresponding to the bit at a given position. A bit equal to 1 expresses the presence of a protein in a particular molecular feature. The first descriptor (CC1rs) used in the developed model, expressed as serine protease (eq 1), indicated that the presence of serine protease affected the value of the zeta potential (ζ) of the studied PNP (Table 1). According to data,^1e^ CC1r characterizes the stability of coated NPs. Furthermore, the experimental results of Donald et al.^26^ clearly show that the presence of lipase and protease treatment of coated NPs did not restore their reactivity. Functionalization in the presence of lipase and protease affects the stability of corona-coated NPs, which, when intact, prevent hemolytic activity and membrane destruction.^1e^ The second corona descriptor used in the developed nano-QSPR model (eq 1) is related to the alpha-amylase inhibitor (AA-I, Table 1). Interestingly, a previous study by Sreeram et al.^24^ showed that the kinetic study revealed that the presence of protease in an enzyme medium affects the biological activity of amylase, as shown by the decrease in the K_m_ and *V*_max_ values of amylase. Immobilization of the enzyme in CuO-NPs prevents denaturation of amylase and has an excellent affinity for CuO^24^ NP. In other words, the biofunctionalization of CuO-NPs with the protease–amylase complex leads to the stability of the NPs. The third corona descriptor used in the developed nano-QSPR model (eq 1) is expressed by kininogen-1 (Table 1). The study described by Sakulkhu et al.^27^ based on superparamagnetic iron oxide nanoparticles (SPION) with two different polymers (poly(vinyl alcohol) polymer (PVA) and dextran (DX)) showed that proteins such as kininogen-1 adsorbed in NP regardless of the type of material and surface charge (positive, negative, and neutral). However, the authors found that PVA-coated SPIONs with negative and neutral surface charges adsorbed more serum proteins than DX-coated SPIONs, resulting in a longer blood circulation time for PVA-coated NPs than for DX-coated ones.^28^ Finally, the descriptors related to the core and coating used in the developed nano-QSPR model (eq 1) clearly show a correlation between the zeta potential and the size of the PNPs (Table 1). These results are consistent with the literature.^12,15^ For example, the results of Yallapu et al.^1a^ showed no significant change in the particle size of the studied NPs after incubation with human serum (HS). At the same time, the zeta potential of NPs became negative due to the adsorption of human serum.^30^ Furthermore, enhanced internalization and uptake of NPs by C4–2B and Panic-1 cancer cells were observed after incubation with human serum (HS).

In summary, the present study shows that the zeta potential of PNPs can be modeled based on the original structure and not only on the NPs themselves. The developed nano-QSPR model (eq 1) clearly shows that the zeta potential should be described as a function of both the structural properties determined by the core and the coating and the biological medium determined by the formation of the protein corona (eq 1, Table 1, S1, and S2). We hypothesize that the presented nano-QSPR model is a step in the development of more sophisticated machine-learning models that support the control and/or design of the stability of NPs in a given medium.

## 3 Conclusions

The identification of core, surface, and biological system features that prevent or induce PNP toxicity would further enhance the ability of nanomaterial experimenters and/or designers to produce safe and smartly targeted nanosystems for various industrial applications. At the same time, it allows them to identify existing high-risk NP–protein corona complexes. Based on these challenges, we identified key parameters that can indirectly determine the biodistribution, uptake, opsonization, kinetics, and toxicity of NPs. In the present study, the 20 PNP structures were characterized by (1) 33 core descriptors, (2) 34 coating descriptors that describe the NP structure itself, and (3) 80 corona descriptors that describe their protein corona fingerprints (PC-FP). The nano-QSPR model (eq 1) based on GA-PLS describes the quantitative relationship between the structure of the PNP, the formation of the value of the protein corona, and the value of the zeta potential (ζ). The developed model GA-PLS is characterized by high external predictability (*Q*^2^_EXT_ = 89%) and a coefficient of determination of covariance *R*^2^ = 96%. Thus, the developed nano-QSPR model is proof of the concept that the zeta potential (ζ) of PNP is determined by both nanostructure features (intrinsic properties described by core and coating descriptors) and protein corona in a given medium (i.e., extrinsic properties described by corona descriptors). Knowledge of how the structure of the original NPs, in combination with surface functionalization and their PC-FPs, affects the potential toxicity of NPs in a given medium may improve understanding of the relationship between the particle surface and particle–cell interactions.

In summary, identifying both the surface properties and the biological medium that are determined by protein corona formation is critical for developing predictive models that describe the stability and toxicity of NPs in real-time. Therefore, the zeta potential of the PNPs was modeled using the original structure of the NPs themselves. The coating and corona descriptors should be considered to make nano-QSPR models suitable for predicting the uptake of nanoparticles (NPs) or for evaluating their potential risk in a real environment. Therefore, nano-QSPR models suitable for predicting the uptake of nanoparticles or evaluating their potential risk in a real environment should include core and coating features. This will enable reliable machine-learning-based predictions that will be of great benefit in the development of safe and sustainable nanomaterials with reduced toxicity.

## 4 Materials and Methods

### 4.1 Nanoparticles and Characterization

The data set for the nano-QSPR model with experimental values of the zeta potential (end point) for 41 polymeric nanoparticles (PNP) was taken from the literature.^14^ Due to (1) part of polymeric nanoparticles (PNP) is in the form of uncoated (nonfunctionalized) NP (sample ID: 1, 2, 3, 4, 6, 7, 8, Table S1) and (2) some of them do not have a zeta potential value (sample IDs: 1, 1A, 1B, 1C, 2, 6, Table S1), the final quantitative relationship between physicochemical properties and biological media was prepared for 20 selected structures. The selected PNPs were characterized by seven different polymers in the size range of 70 to 560 nm. The selected PNPs were characterized by seven different polymers in the size range of 70 to 560 nm. PNPs are characterized by (1) polystyrene (PS), (2) polylactic acid (PLA), (3) polylactic acid-*co*-glycolic acid (PLGA), (4) poly-ε-caprolactone (PCL), (5) polyisobutyl cyanoacrylate (PIBCA), (6) polyhexadecyl cyanoacrylate (NIPAM/BAM), and (7) polyglycidyl methacrylate (PGMA). These PNPs were coated with 7 functional groups, such as carboxyl functions, amine functional, polyglycerol, PEG, dido-decyl dimethylammonium, dextran, and heparin (Supporting Information, Table S1). The final coated structures (20 PNP) are surrounded by 80 different protein compositions, mainly fibrinogen, albumin, etc. (Supporting Information, Table S1). The protein descriptors were divided into two groups indicated by binary values (0 and 1): Group 1, where nanoparticle protein adsorption was present, and Group 0, where no protein adsorption was observed (Supporting Information, Table S1).

### 4.2 Database of Descriptors

To obtain numerical variables that characterize chemical structures, molecular models of all selected polymers (cores) and functional groups (coatings) were created using MOLDEN^31^ software. Each polymer was created in dimeric form. The coating was created as a monomer, and the proteins were described by descriptors called molecular fingerprints (FPs). Geometry optimization was performed for all collected molecular structures using the Gaussian 09 package^32^ with the functionality of B3LYP with a 6-31G basis set. The optimized molecular structures were confirmed by vibrational analysis to ensure an energy-minimized structure with no imaginary frequency. Geometry optimization is useful for extracting the energy-minimized equilibrium conformation, which allows full 3D parameters such as WHIM for descriptor calculations. DRAGON^33^ software was used to calculate molecular descriptors for nanomaterials consisting of cores, coatings, and proteins. Polymeric nanomaterials were characterized by a set of 148 descriptors by adding a subset of each. The final database of descriptors was developed and divided into 33 descriptors for cores, 35 descriptors for coatings, and 80 different proteins (Supporting Information, Tables S1 and S2).

### 4.3 Data Set Splitting

To perform partitioning, the data set of 20 PNP was sorted by the increasing value of the end point (i.e., the zeta potential (ζ)). The data set (Table 1) was divided into two groups: the training group (data to be used to develop a nano-QSPR model, *n* = 14) and the validation group (data to be used to validate the predictive capacity of the model, *k* = 6).^34,35^ Based on these studies,^36^ our model would provide a better prediction with potential data sets, despite a smaller number of basis sets. To ensure a balanced distribution, every third PNP was selected for the validation set (V), and the remaining samples were included in the training set (T) (see Table 1).

### 4.4 Development of the GA-PLS Model

The nano-QSPR model was developed based on a set of intrinsic and extrinsic descriptors calculated for the core, coating, and corona of 20 PNP using the partial least-squares method (PLS).^37−39^ The major advantage of this method is that it converts the original descriptor into latent vectors (LVs) and uses latent vectors that are multicollinear with dependent and independent variables for regression. In this scenario, it is possible to compress the structural information into a smaller number of variables.^40^ To find the most relevant set of core, coating, and corona descriptors in PLS modification, the genetic algorithm (GA-PLS)^38,41^ was used. The results derived from GA-PLS were then used to find a more comprehensive explanation, and it is a random search method to obtain optimized results. In the present study, the value of the end point (*y*_*i*_) of the zeta potential (ζ) is described as the independent variable ***x***_**1**_, ***x***_**2**_, ***x***_**3**_, ..., ***x***_***n***_ using different combinations of primary descriptors, which were previously automatically scaled, as indicated in eq 2.
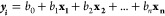
2The nano-QSPR model was validated against the Organization for Economic Cooperation and Development (OECD)standard principles.^43^ A good fit was determined by calculating the determination coefficient (*R*^2^) and the mean square calibration error (RMSEC) based on the prediction for the training set. The robustness of the model, which describes the stability of the sensitivity of the compounds, was checked with internal validation using the Leave-One-Out cross-validation algorithm (LOO).^44^ The robustness of the model was expressed by the cross-validation coefficient (*Q*^2^_CV_) and the root-mean-square cross-validation (RMSE_CV_). Furthermore, the predictive ability of the model was evaluated by calculating the external validation coefficient (*Q*^2^_Ext_) and the root-mean-square error of prediction (RMSE_P_).^38^ All statistical values were calculated according to the formulas summarized in the Supporting Information (Table S4).

The Williams plot consists of standardized residuals versus leverage values used to check and visualize the range (AD) of the QSPR models. According to this formula: *h*_*i*_ = ***X***_*i*_^T^ (***X***^T^***X***), the leverage value (*h*) for the *i*th compound represents the distance of the chemical structure of this compound from the model. A high leverage value (*h*) can strengthen the model if the compounds are included in the training set. The leverage value of the predicted compound shows the values that indicate whether the compound is interpolated or whether the results are extrapolated. If the chemical value is *h* > h*(critical value), where h* = 3 *pn*^–1^, where *p* is the number of variables plus 1 and *n* is the number of compounds in the training set. This means that the model is extrapolated when the predicted *Y* outcomes are out of range (i.e., in the case of interpolation), when *h* < h*, if the model of the chemical’s predicted *Y* outcomes is less reliable than other predictions.^45^ This approach facilitates the visualization of outliers and/or compounds with high leverage and the standardization of the remaining units larger than 3 standard deviation units.

## Supporting Information Available

Characterization of polymeric nanoparticles and corona (Tables S1 and S2), calculated descriptors for the core, coating, and corona (Table S3), data used for the nano-QSPR model (Table S4), and statistical parameters used in the study (Table S5) (XLSX)

## Abbreviations

PC: Protein corona
PN: Periodic number
QSPR: Quantitative structure–property relationship
NPs: Nanoparticles
PNPs: Polymeric nanoparticles
